# RELATIONSHIP BETWEEN STRESS AND PREDEATH GRIEF IN FAMILY CAREGIVERS OF PERSONS WITH DEMENTIA

**DOI:** 10.1093/geroni/igae098.1511

**Published:** 2024-12-31

**Authors:** Lauren Elliott, Sarah Sparks, Sydnie Schneider, Elisabeth McLean, Carol Fadalla, Volker Neugebauer, Jonathan Singer

**Affiliations:** Texas Tech University, Lubbock, Texas, United States; Texas Tech University, Lubbock, Texas, United States; Texas Tech University, Lubbock, Texas, United States; Texas Tech University, Lubbock, Texas, United States; Texas Tech University, Lubbock, Texas, United States; Texas Tech University Health Sciences Center, Lubbock, Texas, United States; Texas Tech University, Lubbock, Texas, United States

## Abstract

Being a family caregiver of a person with Alzheimer’s Disease or Alzheimer’s Disease Related Dementias (AD/ADRD) has been associated with chronic stress. Family caregivers likely experience increased rates of pre-death grief (PDG; grief symptoms that develop while an individual with a life-limiting illness is still living), given the trajectory and terminal nature associated with an AD/ADRD diagnosis. Higher levels of PDG in family caregivers is a robust predictor of depressive symptoms and prolonged grief disorder following the loss. Stress has been extensively studied within this population, but previous studies have failed to link stress to pre-death grief. Data from 65 family caregivers of individuals with AD/ADRD were analyzed (Mage = 68.68, SDage = 11.89). Participants completed questionnaires regarding pre-death grief symptoms (Prolonged Grief—12; PG-12) and subjective stress (Perceived Stress Scale; PSS). Consistent with previous research, family caregivers reported moderate levels of stress (PSS; M = 17.88, SD = 6.42) and pre-death grief (PG-12; M = 27.68, SD = 7.89). A bivariate correlation revealed a significant, positive relationship between stress and pre-death grief (r =.603, p <.001). Thus, family caregivers reporting higher levels of stress were experiencing heightened pre-death grief symptoms. Evaluating subjective stress in family caregivers is a time-efficient, non-invasive way to identify individuals at increased risk for psychosocial dysfunction. Interventions targeting stress in family caregivers may have beneficial downstream effects on pre-death grief before the loss, which may decrease the risk of developing prolonged grief disorder following the loss.

